# Brain signaling systems in the Type 2 diabetes and metabolic syndrome: promising target to treat and prevent these diseases

**DOI:** 10.4155/fso.15.23

**Published:** 2015-11-01

**Authors:** Alexander O Shpakov, Kira V Derkach, Lev M Berstein

**Affiliations:** 1 Laboratory of Molecular Endocrinology & Neurochemistry, I.M. Sechenov Institute of Evolutionary Physiology & Biochemistry, Russian Academy of Sciences, Thorez av. 44, 194223, St. Petersburg, Russia; 2 Laboratory of Oncoendocrinology, N.N. Petrov Research Institute of Oncology, St. Petersburg, Russia

**Keywords:** brain, bromocryptine, diabetes mellitus, insulin, leptin, melanocortin, metabolic syndrome, neurodegeneration, signaling systems, treatment

## Abstract

The changes in the brain signaling systems play an important role in etiology and pathogenesis of Type 2 diabetes mellitus (T2DM) and metabolic syndrome (MS), being a possible cause of these diseases. Therefore, their restoration at the early stages of T2DM and MS can be regarded as a promising way to treat and prevent these diseases and their complications. The data on the functional state of the brain signaling systems regulated by insulin, IGF-1, leptin, dopamine, serotonin, melanocortins and glucagon-like peptide-1, in T2DM and MS, are analyzed. The pharmacological approaches to restoration of these systems and improvement of insulin sensitivity, energy expenditure, lipid metabolism, and to prevent diabetic complications are discussed.

## Abstract (lay abstract)

Currently, more than 30% of the populations worldwide are overweight and have metabolic dysfunctions that without the appropriate treatment would go over to severe Type 2 diabetes mellitus (T2DM). At the early stages of T2DM significant changes occur in the brain signaling systems, resulting in the impairment of metabolism and the functions of nervous, cardiovascular and the other systems. Restoration of the brain signaling is regarded as a promising way to treat and prevent diabetic pathology. In the review the changes in the brain signaling systems in T2DM and the pharmacological approaches to their restoration are discussed.

**Figure 1.**
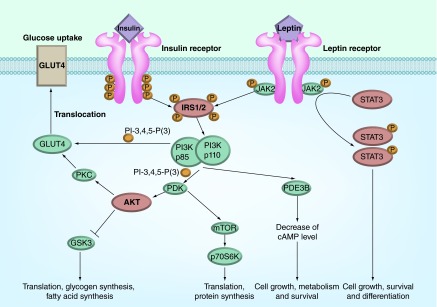
**Insulin and leptin signaling.** AKT: Protein kinase B; GLUT4: Insulin-regulated glucose transporter of the type 4; GSK3: Glycogen synthase kinase 3; IRS1/2: Insulin receptor substrates 1 and 2; JAK2: Janus kinase-2; mTOR: Mammalian target of rapamycin; p85-PI3K and p110-PI3K: Regulatory (p85) and catalytic (p110) subunits of heterodimeric p85/p110 phosphatidylinositol 3-kinase; PDE3B: Phosphodiesterase of the subtype 3B; PDK: Phosphoinositide-dependent kinase; PI-3,4,5-P(3): Phosphatidylinositol 3,4,5-triphosphate; PKC: Protein kinase C; STAT3: Signal transducer and activator of transcription of the type 3.

**Figure 2.**
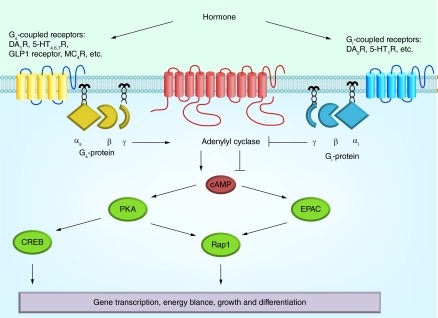
**The hormone-sensitive adenylyl cyclase signaling system.** α_s_βγ and α_i_βγ: Heterotrimeric G_s_- and G_i_-proteins; 5-HT_1,4,6,7_R: 5-hydroxytryptamine receptors of the types 1, 4, 6 and 7; cAMP: 3′,5′-cyclic adenosine monophosphate; CREB: cAMP response element-binding; D_1,2_DAR: Dopamine receptors of the types 1 and 2; EPAC: cAMP-responsive Rap1 guanine nucleotide exchange factor; GLP-1: Glucagon-like peptide-1; MC_4_R: Melanocortin receptor of the type 4; PKA: Protein kinase A; Rap1: Ras-related protein 1.

Currently, more than 30% of the populations worldwide are overweight and have metabolic disorders. Without treatment and prevention of these disorders, they would go over to prediabetes that is characterized by decreased insulin sensitivity and then, in accordance with the adverse scenario, to overt Type 2 diabetes mellitus (T2DM) and metabolic syndrome (MS) commonly associated with cardiovascular, nervous, endocrine and other diseases. One of the most promising ways to prevent overt T2DM and MS and their complications is to begin treating patients with early stages of insulin resistance and dyslipidemia when metabolic disorders are still reversible.

The main factors responsible for the development of T2DM and MS are insulin resistance, dysfunctions of pancreatic β-cells, hyperglycemia, the formation of advanced glycation end products, oxidative stress, mitochondrial dysfunctions, dyslipidemia, lipotoxicity and alterations in the hormonal signaling systems both in the CNS and the periphery [1–6]. The hormonal dysfunctions are sometimes referred to either as primary, or secondary, the former being one of the causes of T2DM and MS, while the latter – the consequence of cell and tissue damages induced by oxidative stress and lipotoxicity [7–10].

The brain signaling systems regulated by insulin, IGF-1, leptin, neuropeptides, monoamines and neurotrophic factors are of great interest in the study of etiology and pathogenesis of T2DM and MS. Despite the role of the above systems in the development of these diseases is not quite clear yet, there is enough evidence to suggest that changes occurring in them can be a trigger for T2DM and MS [11–15]. The most critical changes occur in the signaling systems of hypothalamus, an integrator of regulatory processes in the brain and in the periphery. The hypothalamic signaling systems are involved in regulation of insulin sensitivity, glucose and lipid metabolism, feeding behavior, as well as in control of functions of the nervous, endocrine and cardiovascular systems [9,16–21]. The weakening of the response of POMC/cocaine and amphetamine-regulated transcript neurons located in the arcuate nucleus (ARC) of hypothalamus to insulin, leptin, ghrelin and GLP-1 leads to decreased production of melanocortins, to the impaired melanocortin signaling system in the paraventricular nucleus (PVN) of hypothalamus, which brings about the changes of neural circuits involving the lateral hypothalamic area and the ventral tegmental area responsible for food intake behavior and energy expenditure [22–25]. Simultaneously, the activity of AGRP/NPY neurons located in the ARC is also changed, which leads to the increased food intake. Since the mesolimbic dopaminergic system is involved in the response to food rewards, the abnormalities in this system and in its regulation by leptin and ghrelin contribute significantly to eating disorders [26,27]. The important role in deregulation of the feeding behavior is ascribed to the changes in the circadian rhythm, which induced the disturbances in synchronization of the release of serotonin (5-HT), dopamine (DA) and other neurotransmitters and provoked the dysfunctions of neural circuit responsible for food rewards [28–30].

The weakening of functions of the brain hormonal systems can be caused by reduction of the levels of signal molecules due to impairment of their synthesis, secretion and transport within neuronal cells, and by the alterations in expression and activity of proteins, the components of these systems. Initially, the changes can occur in a single signaling cascade in specific brain area and then extend to the other central signaling systems, which leads to deregulation of energy homeostasis and to decrease of insulin sensitivity, and finally to prediabetes state [31–36]. Restoration of the brain signaling systems at the stage of prediabetes can induce normalization of the central regulation of the peripheral metabolism and prevent the transition of prediabetes into overt T2DM and MS [37]. To achieve this, it is necessary to detect the point violations in the brain signaling systems at the early stages of diabetic pathology, to decipher their molecular mechanisms, and to reveal changes in the biochemical and physiological processes induced by the disturbances in these systems. In the case of overt T2DM and MS the changes occur in many signaling systems as a result of a comprehensive damage of different brain areas and neural circuits due to the inflammation, mitochondrial dysfunctions and endoplasmic reticulum stress [38]. To cope with these tasks, the systemic approach to restoration of the brain signaling must be developed based on the correction of metabolic dysfunctions and the improvement of functions of the brain signaling network.

In this review the brain signaling systems regulated by insulin, IGF-1, leptin, DA, 5-HT, melanocortins and GLP-1 are analyzed. The role of these systems in etiology and pathogenesis of T2DM and MS has been established and now they are considered to be the most suitable targets in the therapy of these diseases. Meanwhile, in patients and experimental animals with T2DM and MS the functional activity of the brain signaling systems regulated by ghrelin, NPY, acetylcholine, adrenergic agonists, γ-aminobutyric acid and glutamate was also shown to be significantly changed, but there are, however, little data that the pharmacological restoration of these systems significantly affects the physiological and biochemical processes in the CNS and periphery impaired in metabolic disorders [39–49].

## Brain insulin & IGF-1 signaling

### Insulin/IGF-1 signaling system in the CNS

In the brain insulin and insulin receptor (IR) were discovered more than 30 years ago [50–52]. Since then, a lot of evidence to support the key role of the brain insulin signaling system in regulation of physiological and biochemical processes in the CNS and in the periphery, and in control of feeding behavior and peripheral metabolism was obtained [53–56]. IGF-1 and its receptor (IGF-1R) were also found in the brain, and it was shown that IGF-1 signaling system is involved in control of neurogenesis and synaptogenesis and interacts with other central signaling systems [57–59]. The insulin and IGF-1 signaling systems in the CNS and in the periphery have a considerable similarity in structural and functional organization, but their regulatory mechanisms may be different. This can be explained by the difference in the pattern of systems components, in their microenvironment and localization in cell, and concerning the insulin system, in the neuronal and non-neuronal types of IR [56].

Insulin and IGF-1 belonging to the insulin peptides family bind to α_2_β_2_-heterotetrameric IR and IGF-1R [60]. The extracellular α-subunits of these receptors contain the ligand binding site, whereas the intracellular portion of their β-subunits contains the highly conserved tyrosine kinase domain. The binding with hormone induces the tyrosine kinase activity of receptor, which leads to tyrosine phosphorylation of insulin receptor substrates-1 and -2 (IRS-1 and IRS-2) [61,62]. The IRS-1 mediates the regulatory effects of insulin and IGF-1 on the growth and metabolic processes mainly in the peripheral tissues, whereas IRS-2 is preferably responsible for the effects of hormones in the CNS, including the control of neuronal growth and differentiation, the hypothalamic regulation of feeding behavior, body weight, glucose homeostasis and reproductive functions [63]. Phosphorylated IRS-1 and IRS-2 activate a large number of proteins that contain SH2-domains specifically interacting with phosphotyrosines, such as the enzymes – PI3K, protein phosphotyrosine phosphatase SHP2, and nonreceptor tyrosine kinase Fyn, and the adapter proteins – suppressor of cytokine signaling, growth factor receptor-bound protein 2, Nck-protein and others [63,64]. This leads to activation of many downstream signaling cascades involved in the regulation of insulin/IGF-1-dependent transcription factors which control growth, differentiation, apoptosis and the other cell processes.

The 3-phosphoinositide pathway includes heterodimeric p85/p110-PI3K catalyzing the synthesis of phosphatidylinositol-3,4,5-triphosphate, and two serine/threonine protein kinases, PDK1 and protein kinase B (AKT-kinase) (Figure 1). The binding of pleckstrin homology domains of PDK1 and AKT-kinase with phosphatidylinositol-3,4,5-triphosphate leads to translocation of enzymes into the plasma membrane, where AKT-kinase is phosphorylated by PDK1 and mammalian target of rapamycin (mTOR) Complex 2 on Thr^308^ and Ser^473^ residues, respectively [65,66]. The activation of AKT-kinase leads to translocation of insulin-dependent transporter GLUT4 into the plasma membrane and increases the glucose uptake. The other targets of AKT-kinase are mTOR Complex 1, GSK3, BAD, FKHRL1 and FBP-1. The phosphorylation of GSK3 leads to its inhibition and blocks the negative influence of GSK3 phosphorylation on glycogen synthase, a key enzyme of glycogen production. Alongside, the inhibition of GSK3 changes the activity of many transcriptional factors, such as NF-κB, forkhead transcriptional factors FOX3a and FOXO1a, Bcl-2 associated death promoter, regulating the gene expression, apoptosis and cell viability [67].

### The dysfunctions of insulin/IGF-1 signaling in diabetes mellitus

In the Type 1 diabetes mellitus (T1DM) with a strong insulin deficiency, despite the increased hormone transport across the blood–brain barrier (BBB), the concentration of insulin in the brain is nevertheless very low. In T2DM and MS with normal or elevated insulin concentration in the blood the transport of insulin through the BBB is attenuated, resulting in the decrease of brain insulin level [68–74]. As a consequence, functional activity of the brain insulin signaling system is attenuated, which negatively affects the central control of peripheral metabolism and thus enhances the severity of metabolic disorders and insulin resistance.

According to some authors, the concentration of total IGF-1 in T2DM and MS in the bloodstream did not change significantly [75,76]. However, in long-term T2DM with poor glycemic control the concentration of bioavailable IGF-1 was reduced partially due to doubling of the level of IGFBP1 [75,77]. It should be noted that in obese patients with weak insulin resistance the free IGF-1 level increased insignificantly due to the lowered IGFBP1 level, while total IGF-1 level was maintained. With increasing insulin resistance, in overt T2DM, the IGFBP1 level increased, which caused a significant decrease in free IGF-1 concentration [78]. Appreciable contribution to reduction of free IGF-1 level was made due to increasing IGFBP3 level [79]. One of the causes of increase in the IGFBP levels in the bloodstream is a nonenzymatic glycosylation of these proteins in hyperglycemic conditions, as the severity and duration of hyperglycemia and insulin resistance are positively correlated with the increase of IGFBP levels [80,81]. Additionally, the relationship was established between the increased IGFBP level and the decreased IGF-1/IGFBP ratio, on the one hand, and the development of Alzheimer's disease (AD) designated sometimes also as the Type 3 diabetes, on the other [82]. The conclusion was made that the decrease of free IGF-1 level is one of the factors of the weakening of the brain IGF-1 signaling in prediabetes, early T2DM and AD.

Despite the fact that the decrease of insulin and IGF-1 levels in the brain makes a significant contribution to reduction of the activity of insulin/IGF-1 signaling systems in T2DM and MS, the key role is given to abnormalities in these systems caused by oxidative stress, lipotoxicity and increased production of proinflammatory and proapoptotic factors [16,83–86]. The deregulation of the brain insulin/IGF-1 signaling pathways influences energy metabolism and insulin resistance, and results in triggering pathological changes in the nervous and neuroendocrine systems [87–89].

The role of insulin resistance in etiology and pathogenesis of T2DM, MS and AD is currently not in doubt, but there arise many questions concerning the relationship between the insulin resistance in different brain areas and in the peripheral tissues [15,56,90]. It is not clear whether or not they develop in parallel and for how long they exist independent of each other, causing various forms of pathology [15,91–94]. If there is a functional relationship between them, is insulin resistance in the brain capable of inducing insulin resistance in the periphery and to cause T2DM?

Recently, the hypothesis of central genesis of T2DM has been developed intensively. According to it, one of the trigger mechanisms of T2DM is the central insulin resistance [11–15]. The identification of the relationship between the insulin resistances in the CNS and in the periphery offers great opportunities for the treatment of T2DM at the early stages. In humans, insulin resistance takes a long time, usually 10–15 years, to develop before there appear signs of T2DM. The data from clinical trials show that a significant number of patients with the decreased insulin sensitivity (prediabetes) in the brain demonstrated the early stages of AD [95,96]. Later, due to prolonged central insulin resistance, some patients have the late stages of AD with dementia, but without transition of prediabetes into T2DM, while the other AD patients have the signs of overt T2DM. In the cortex and hippocampus of patients with AD the insulin and IGF-1 resistance caused by the weakening of the signaling pathways IR/IRS-1/PI3K and IGF-1R/IRS-2/PI3K was detected [88].

Using the MS and T2DM models, the evidences were obtained that a decrease in activity of the brain insulin system led to an impairment of energy homeostasis and peripheral insulin sensitivity. The neuron-specific IR knockout mice (NIRKO) had hyperphagia, the increased body weight, hyperglycemia, moderate insulin resistance, and the increased levels of insulin, leptin and triglycerides [83,85]. The inactivation of IR in the ARC of the hypothalamus with antisense oligonucleotides led to insulin resistance in the liver and, consequently, to a weakening of the ability of insulin to suppress the peripheral glucose production by hepatocytes, inducing the moderate hyperglycemia and dyslipidemia [16,84]. In the brain of NIRKO-mice insulin did not activate PI3K and AKT-kinase, which increased the phosphorylation of microtubule-associated Tau-protein [85]. In obese mice, hyperphosphorylation of Tau-protein was associated with the resistance of neuronal cells to insulin and IGF-1 [97]. Site-specific hyperphosphorylation of Tau-protein is the main mechanism mediating the link between central insulin/IGF-1 resistance and neurodegenerative changes [97,98]. The increased phosphorylation of Tau-protein in T2DM and AD was independent of peripheral insulin resistance, hyperinsulinemia and hyperglycemia, indicating the key role of central mechanisms in the regulation of this process [99]. The expression of gene encoding the neuron-specific IR in the brain of mice lacking both IR and GLUT4 increased survival of animals and improved the energy metabolism, despite the GLUT4 deficiency [100].

In the cortex and hippocampus of hamsters with MS induced by fructose diet a decrease of activity of IR, IRS-1 and AKT-kinase was showed. The main cause for this was the increase of the expression of PTP1B that dephosphorylates IR and IRS-1, breaking the transduction of insulin/IGF-1 signals through the 3-phosphoinositide pathway [73]. Other authors revealed no changes in IR functions in the brain of rats with spontaneous T2DM, but detected the decreased expression and altered binding properties of IGF-1R, which, similarly to the case of the reduced IR activity, led to a decrease of AKT-kinase activity [101].

An important role in the disturbances of the brain insulin signaling system has a decrease in activity or shutdown of IRS-proteins and PI3K resulting in insulin resistance, hyperphagia, carbohydrate and lipid metabolism abnormalities, which leads to T2DM and MS [102–104]. The deletion of the *irs2* gene in the hypothalamic nuclei of mice induced the increase of appetite, fat and muscles weight, linear growth, and eventually led to insulin resistance. The signs of overt T2DM were identified in mice at the age of 6–10 months [105]. The intracerebral administration of PI3K inhibitor reduced the phosphorylation of AKT-kinase in the hypothalamus and induced an increase in the insulin resistance [103]. The same result was obtained due to dysregulation of the 3-phosphoinositide pathway by melatonin, which is responsible for generation of circadian rhythms and through the brain 3-phosphoinositide pathways inhibited gluconeogenesis in the liver [106]. The treatment of mice with antagonists of melatonin receptors also led to insulin resistance, whereas the treatment with melatonin, on the contrary, increased the phosphorylation of AKT-kinase and restored insulin sensitivity. It should be mentioned that the decline in brain melatonin level was observed in many patients with prediabetes and T2DM [107]. Moreover, removal of the epiphysis producing melatonin increased gluconeogenesis and provoked insulin resistance in the liver [108]. These data indicate a close relationship between circadian rhythm disorders and insulin resistance, and demonstrate participation of the brain melatonin signaling in control of peripheral insulin sensitivity [109].

### The approaches to improve the brain insulin/IGF-1 signaling

The most promising approach to restore the activity of brain insulin signaling system in T2DM and MS is to increase insulin level in the CNS. Injectable insulin is not very reasonable in this case, since the transport of peripheral insulin into the brain in the conditions of insulin resistance is impaired. The intracerebroventricular administration of insulin can be used only in experimental conditions. Therefore, the best way is intranasal route of insulin delivery, as it leads to an increase of intracerebral concentration of hormone [110–112]. This method is easily reproducible and requires no special equipment. Of note, in the recent years, intranasal route has been widely used for delivery of many hormones [112,113].

In the recent years the intranasal insulin (I-I) has been widely used to correct AD, posttraumatic stress disorders and other cognitive dysfunctions [114–120]. Meanwhile, the efficiency of I-I therapy in the treatment of T2DM and MS is not so obvious, which is largely due to some gaps in the knowledge concerning the mechanisms of I-I action on the brain signaling, insulin sensitivity and peripheral metabolism. Despite this, there are encouraging evidences that brain insulin resistance can at least partially be overcome by I-I, as shown in the clinic and under experimental conditions, which is important in the treatment of both AD and diabetic pathology that are characterized by the decreased insulin sensitivity in the CNS [121].

According to our data long-term I-I treatment of rats with the neonatal and the high-fat diet (HFD) models of T2DM improved glycemic control and restored the insulin sensitivity [122,123]. In our view this depends to a great extent on the ability of I-I to restore activity of the adenylyl cyclase (AC) signaling system regulated by monoamines and peptide hormones in the brain and peripheral tissues [122–124]. It was shown by the other authors that the treatment of rats with I-I increased insulin sensitivity in adipocytes and suppressed the lipolysis in the white adipose tissue, improving the lipid metabolism [125]. These data give basis for the conclusion that I-I treatment can prevent or attenuate the complications of T2DM, including diabetic encephalopathy and cardiomyopathy. Note that I-I treatment of rats with T1DM also restored the functions of the AC signaling system in the brain and in the periphery, and improved the cognitive functions [126,127].

The expected prospects in developing the drugs to increase the brain insulin levels are associated with the use of inhibitors of the activity of insulin degrading enzyme (IDE), which destroys the hormone molecule [128,129]. However, no commercial drugs that are selective IDE inhibitors have been available yet. Neither the molecular mechanisms of their action have been studied in detail, because these inhibitors influence the signaling pathways regulated by different hormones [129]. At the same time, in the experimental conditions IDE inhibitors had a pronounced hypoglycemic effect. Recently, highly potent inhibitor 6bK based on the 20-membered macrocycle structure was developed, and it substantially improved glucose tolerance in lean and diet-induced obese (DIO) mice [129]. It may be expected that in the coming years selective IDE inhibitors will be one of the most successful drugs to correct the brain insulin signaling in T2DM and MS.

The IGF-1 level in the brain can also be increased to improve the metabolic processes and restore the CNS functions in MS and T2DM, but the available data on application of this approach refer only to experimental models of T1DM [130,131]. The effective doses of intracerebrally administered IGF-1, compared with insulin, were significantly lower, and the effect of IGF-1 increased when it was administered together with insulin [131]. It can be assumed that the increase of the levels of insulin and IGF-1 provides potentiation of their stimulating effect on the 3-phosphoinositide pathway in neuronal cells. This makes it necessary to use co-administration of IGF-1 and insulin to improve central and peripheral insulin sensitivity in T2DM and MS.

In the last few years, the data on the therapeutic effect of biguanide metformin on the CNS in T2DM and MS, as well as its positive influence on the brain insulin signaling were obtained [132]. Currently, metformin and its analogs are the main drugs used in the treatment of patients with T2DM and its complications, including cardiovascular disorders and cancer [133]. In the CNS, metformin exhibits the antioxidant and neuroprotective effects, prevents the neurodegenerative changes and restores the neuronal signaling network. In the rat models of T2DM the four-week metformin treatment led to a weakening of oxidative stress in the CNS [134]. This drug inhibited the apoptotic neurodegeneration induced by etoposide and ethanol in rat cortical neurons [135,136]. Metformin stimulated the AMPK-dependent processing of β-amyloid peptide and activated phosphatase PP2A which dephosphorylates Tau-protein in neuronal cells, thereby reducing the accumulation of β-amyloid peptide and the phosphorylation of Tau-protein, and preventing the neurodegeneration [137–139]. Co-administration of metformin and insulin caused the potentiation of their inhibitory effect on the formation of amyloid fibrils in neurons [140]. However, the efficacy of the inhibitory effect of metformin on the neurodegeneration was strongly dependent on the severity of metabolic disorders, the models of neuronal insulin resistance and other factors, because in some cases its effect was significantly reduced [132,140]. All this should be taken into consideration in the treatment of T2DM-associated neurodegenerative diseases with metformin.

Another promising and rather new approach used for restoration of the brain insulin/IGF-1 signaling is inhibition of PTP1B, a negative regulator of the signaling, that dephosphorylates and inactivates both receptors and IRS-proteins [141]. The PTP1B has a significant impact on the development of insulin resistance and metabolic disorders [21]. The administration of Trodusquemine and Claramine, the specific PTP1B inhibitors, into the brain suppressed PTP1B activity in hypothalamic neurons and activated insulin signaling in them. This effect was observed in mice with HFD-induced T2DM and in mutant mice with the central and peripheral insulin resistance [142–144]. Trodusquemine and Claramine penetrated the BBB quite easily. Therefore, injected intraperitoneally, they inhibited the PTP1B activity in the CNS and in the periphery, thereby restoring the activity of insulin signaling system in the brain, liver, skeletal muscles, and the other organs and tissues. In addition, these inhibitors prevented the negative influence of PTP1B on leptin signaling pathways involved in the central regulation of energy expenditure and insulin resistance [145].

## Leptin signaling system

Leptin, product of the *ob* gene, is synthesized in adipocytes and transported through the BBB into the brain, and specifically binds to leptin receptors belonging to the family of cytokine receptors [146]. The highest density of leptin receptors is characteristic of the hypothalamus, but they were also detected in the cortex, thalamus, cerebellum, olfactory bulb and choroid plexus [147–149]. The binding of leptin with functionally active long form of leptin receptor OBRb induces the activation of nonreceptor tyrosine kinase JAK2 (Figure 1). The JAK2 kinase phosphorylates the residues Tyr^985^, Tyr^1077^ and Tyr^1138^ located in the intracellular domain of leptin-activated receptor OBRb, and the phosphotyrosine residues provide the interaction between phosphorylated OBRb and the SH2-domain-containing proteins. The phosphorylated residues Tyr^1077^ and Tyr^1138^ interact with the transcription factors STAT5 and STAT3, while the phosphorylated residue Tyr^985^ interact with SH2-containing protein tyrosine phosphatase-2 (SHP2), activating the kinases ERK1/2 [146,150,151]. The activation of the transcription factors STAT3 and STAT5 provokes transcription of the STAT3/STAT5-dependent genes. The formation of the ternary complex consisting of leptin-activated receptor OBRb, phosphorylated JAK2 kinase and SH2-containing protein SH2B1 leads to tyrosine phosphorylation of IRS-proteins and further activation of PI3K and AKT-kinase [146,152,153].

The ability of leptin to activate the 3-phosphoinositide pathway that is involved in the regulation of the metabolic and growth processes in neuronal cells, and in the interaction of the leptin signaling system with the brain systems regulated by insulin, IGF-1, melanocortins, NPY and GLP-1, point to the key role of leptin in control of feeding behavior, energy expenditure, insulin resistance, neurogenesis, neuroprotection and synaptic plasticity [85,154–162]. The influence of leptin on the NPY and melanocortin systems of the hypothalamus is realized due to the ability of this hormone regulating the synthesis and secretion of NPY, α-MSH and AGRP, endogenous antagonist of the melanocortin receptors, in hypothalamic neurons [154,156,158]. Leptin through the 3-phosphoinositide pathway inhibits the apoptotic processes in neuronal cells and prevents the degradation of dopaminergic neurons caused by toxins [155,163]. Note that the dopaminergic neurons are involved in the effects of leptin on feeding behavior and energetic balance [164]. As a consequence, the decrease of leptin level and of activity of leptin system in the hypothalamus and other brain areas result in imbalance of neuronal interactions, abnormalities in the central regulation of peripheral metabolism, and insulin resistance, which leads to the metabolic, neuroendocrine and neurological disorders, including severe obesity, MS and T2DM [165].

The relationship between the decrease in leptin sensitivity in the brain and the development of insulin resistance was shown in patients with obesity, MS and T2DM having the Gln^223^Agr and Asp^100^Tyr mutations in gene encoding leptin receptor OBRb [35]. The mutations reduced the activity of the receptor OBRb and inhibited the transduction of leptin signal, decreasing insulin sensitivity in neurons. In experimental MS it was shown that intracerebral administration of leptin significantly reduced the insulin doses required to control the blood glucose level, due to the weakening of insulin resistance and the decrease of production of glucagon, a functional antagonist of insulin [166,167]. Since glucose homeostasis is regulated through the 3-phosphoinositide pathway, the latter being the target of insulin and leptin, the interaction between the leptin and insulin signaling systems is realized at the level of PI3K and AKT-kinase in the hypothalamus, and this molecular mechanism mediates the effects of leptin on insulin sensitivity and insulin-dependent glucose uptake [85,157,165].

Another mechanism of leptin action on insulin sensitivity consists in regulatory influence of leptin on hypothalamic melanocortin system. This finds support in the results of investigation where the agonists of the types 3 and 4 melanocortin receptors (MC_3_R and MC_4_R) enhanced the ability of intracerebrally administered leptin to increase glucose utilization and to restore insulin sensitivity and lipid metabolism in mice with hyperglycemia, dyslipidemia and hyperinsulinemia, while their antagonists, on the contrary, prevented these effects of leptin [168]. The intracerebral administration of leptin into mice with T1DM restored the expression of POMC, the precursor of α- and γ-MSH, and the activity of the brain melanocortin system, and, thus, improved glycemic control [166,169].

There are many evidences to support the view that the weakening in activity of the brain leptin signaling system both in diabetic and nondiabetic pathology leads to the neuronal damage, the impaired synaptic plasticity and hormonal sensitivity of hypothalamic neurons [170–174]. Such weakening can be provoked by the central leptin deficiency, the altered activity of leptin receptor and IRS-proteins, and the decreased response of hypothalamic neurons to insulin [175,176]. It was shown that the leptin deficiency and the leptin resistance in the CNS were involved in the impairment of cognitive functions in T2DM, MS and also in experimental type 1 DM [171,174,177,178]. Since leptin stimulates the transcription of genes encoding IR and IRS-proteins, a decrease of its regulatory effects at the early stages of insulin resistance leads to weakening of function of the brain insulin system and exacerbates insulin resistance. This process, a kind of avalanche by nature, gives rise to the development of combined insulin and leptin resistance in neuronal cells, which leads to deterioration of the metabolic and neurodegenerative disorders. The restoration of the brain leptin system with intracerebrally injected leptin improved the neuronal plasticity and suppressed the neurodegenerative processes in the animals with MS and T2DM [166,169].

Despite great potential of the approach based on the restoration of the brain leptin signaling in metabolic disorders, currently leptin is used to a limited extent in clinic to treat and prevent MS and T2DM. This is due to the development of leptin resistance, a decrease of the level of endogenous leptin, and inefficiency of leptin therapy in the case of the central leptin signaling being impaired [171,179]. In some cases the replacement therapy with leptin is effective. The daily subcutaneous injections of recombinant leptin led to a reduction of hyperphagia and body weight in children with the severe obesity caused by inactivating mutations in the gene encoding leptin [180]. The leptin therapy gives good results in diabetic and nondiabetic patients with lipodystrophy, who have significantly reduced production of endogenous leptin due to a deficiency of fat mass [181–183]. This treatment prevents the development of overt T2DM, as the restoration of leptin levels normalizes lipid metabolism in the liver and muscles. The combination of leptin therapy and caloric restriction is the appropriate approach to reduce the body weight and fat mass in obese patients, since the caloric restriction alone leads to a significant decrease in the leptin level and suppresses the leptin signaling in the hypothalamus, preventing the weight loss [184]. At the same time, in the last few years many approaches to restore the leptin signaling were developed, and they were quite effective (see the review by Roujeau and co-authors [185]).

A promising approach is the use of leptin administered together with amylin, cholecystokinin and GLP-1, which significantly increase the sensitivity of cells, hypothalamic neurons in particular, to leptin and enhance leptin effect on insulin sensitivity and metabolism [186–188]. The treatment of obese DIO-rats with a combination of leptin and amylin led to a significant decrease in appetite and body weight, while the monotherapy was not enough effective [189,190]. It should be noted that DIO-rats had leptin resistance and this was the main cause of low efficiency of leptin monotherapy. Co-administration of leptin and amylin significantly increased the sensitivity of hypothalamic neurons to leptin, which caused a decrease in appetite and was the mechanism that contributed to the synergistic effect of these hormones [191]. The addition of cholecystokinin to the combination of leptin and amylin considerably increased their therapeutic effect [186]. Co-administration of leptin and the agonists of GLP-1 receptor, exendin-4 in particular, into obese rodents restored leptin sensitivity, normalized feeding behavior and improved the lipid and carbohydrate metabolism [187,188,192,193]. The combination of leptin with clusterin, a ligand for LDL receptor-related protein-2, was also effective and led to the increase of anorectic effect of leptin and its ability to activate the transcription factor STAT3 in hypothalamic neurons [194]. It should be noted that the knockout of the *Stat3* gene or the mutation of STAT3-interacting residues Tyr^1138^ in leptin receptor led to the obesity and hyperphagia [195–197].

Another approach is to apply the peptide fragments corresponding to the C-terminal region 116–122 of leptin, the most effective of which is peptide [D-Leu-4]-OB-3. In C57BL/6J *ob*/*ob* mice this peptide improved the glucose control and insulin sensitivity [198]. On the basis of peptide [D-Leu-4]-OB-3, the drugs effective in intranasal and oral administration were developed whose activity significantly surpassed that of the same peptide when administered parenterally [199,200]. The peptide analogs of leptin easily penetrated the BBB and specifically interacted with leptin receptors in the CNS, activating leptin signaling in the hypothalamus [185].

The increase of leptin sensitivity can be achieved with inhibitors of the phosphatase PTP1B and the suppressor of cytokine signaling 3, the negative regulators of leptin signaling [201–203]. The treatment of DIO-mice with Trodusquemine, an allosteric PTP1B regulator, decreased appetite, body weight and adipose tissue mass, and increased the sensitivity of neurons to leptin and insulin [142]. This effect was due to its ability to prevent the inhibition of JAK2- and STAT3-dependent pathways responsible for anorectic influence of leptin. The compound JTT-551, a highly selective PTP1B inhibitor, also increased leptin signaling in DIO-mice [204]. A single co-administration of leptin and JTT-551 led to the activation of STAT3-dependent pathways in the hypothalamus and suppressed the appetite. The long term, 6-week, co-administration of leptin and JTT-551 into obese animals had a pronounced antidiabetic effect and improved glucose and lipid homeostasis [204]. It was also suggested to use, in addition to PTP1B inhibitors, the regulators of leptin receptor processing, including the inhibitors of endospanin-1 preventing leptin receptor translocation to the plasma membrane [205] and leptin derivatives with increased ability to penetrate the BBB [206,207]. A wide range of pharmacological agents capable of activating the brain leptin system, and a significant importance of this system in control of insulin sensitivity and energy balance allow considering the leptin signaling in the brain as one of the main targets in the treatment of MS and T2DM, including the early stages of these diseases.

## Dopamine signaling system

The neurotransmitter DA controls many physiological functions, including locomotor activity, cognition, emotions, feeding behavior. It also regulates the endocrine, cardiovascular and digestive systems. The cellular effects of DA are realized through five types of DAR, all coupled to the enzyme AC (Figure 2). By binding to the types 1 and 5 DAR, the DA stimulates AC activity, and by binding to the types 2, 3 and 4 DAR inhibits it [208].

With a decrease of the brain DA level and the activity of DA_2_R, the activity of brain dopaminergic system in patients and in experimental animals with MS and T2DM weakened significantly [209,210]. The restoration of activity of the dopaminergic signaling system can be achieved using the selective DA_2_R-agonists that normalize the CNS functions impaired in diabetic pathology, improve the glycemic control and prevent the cardiovascular diseases. The most effective among them is alkaloid bromocryptine (BC), a selective DA_2_R-agonist that activates G_i_ protein-coupled DA_2_R and decreases the intracellular cAMP levels in neuronal cells. The BC inhibits the activity of hypothalamic neurons controlling glucose production and lipid synthesis in the liver and, in addition, activates dopaminergic neurons regulating the insulin sensitivity [210–214]. Historically and up to now, BC had widely used to treat Parkinson's disease and hyperprolactinemia.

The first publication devoted to the hypoglycemic effect of BC (Cycloset) appeared in 1999 [215]. Now there is evidence for high efficiency of BC and its analogs in the treatment of patients with MS and T2DM [210,212,216–223]. The long-term BC therapy of diabetic patients reduced the insulin resistance index and the levels of glycated hemoglobin, triglycerides and LDL-cholesterol, which indicates normalization of the carbohydrate and lipid metabolism [212,216,217,219,222]. The data are available showing the positive effect of BC on the metabolic status in experimental animals with MS and alloxan-induced T1DM [224–226]. The BC treatment of rats with MS normalized the levels of postprandial glucose, insulin and triglycerides [224]. The BC lowered the blood glucose level but had no significant influence on insulin level, neither did cause hypoglycemic episodes adversely affecting the CNS.

The effectiveness of BC influence on glycometabolic parameters was comparable to that of metformin, widely used antidiabetic drug. When co-administered, BC caused the increase of hypoglycemic effect of low-dose insulin and enhanced the glucose-lowering effect of metformin, glipizide and pioglitazone, as demonstrated in clinical trials [211,222,227,228] and in animal models of MS [224,225]. The treatment of patients with T2DM with a combination of metformin (1000 mg/day) and BC (0.8–1.6 mg/day) induced a more pronounced decrease of the levels of fasting and postprandial glucose and glycated hemoglobin than in the monotherapy [228]. Co-administration of BC and metformin to patients with T2DM enhanced the glucose-lowering effect of these drugs, reduced their effective doses and allowed avoiding the side effects [227]. The potentiation of glucose-lowering effect of co-administered BC and glipizide was shown in rats with alloxan T1DM [225].

Proceeding from the clinical and experimental results the assumption was made that BC-induced restoration of insulin resistance and glucose production in the liver were the result of the improvement of the dopaminergic system in hypothalamic neurons involved in control of the central and peripheral insulin sensitivity [212]. It should be mentioned at this point that the brain dopaminergic system, primarily DA_2_R, is involved in control of feeding behavior and energy expenditure, and, therefore, is an important regulator of glucose homeostasis [229]. The ability of BC to restore insulin sensitivity in T2DM can be explained in terms of the hypothesis of central genesis of insulin resistance, which, as said above, is based on the concept of impairments in the dopaminergic and other neurotransmitter systems, regarded as the prime causes of MS and T2DM [11–15].

The abnormalities in the integrative signaling network of the hypothalamus, which include the decreased activity of the dopaminergic signaling system in hypothalamic neurons, the increased noradrenergic and serotonergic signaling in the ventromedial hypothalamus, and the increase of the levels of NPY and corticoliberin in the hypothalamic PVN all led to hyperactivation of the hypothalamic–pituitary–adrenal axis and to release of cortisol, and to the increase of activity of the sympathetic nervous system and its influence on the liver, adipose tissue and the cardiovascular system [230–232]. Further increase of activity of the sympathetic nervous system in the adipose tissue provoked the changes in lipid metabolism, enhanced the secretion of proinflammatory factors and, hence, provoked insulin resistance [233]. The increase of cortisol level and the increased activity of the sympathetic nervous system in the liver enhanced the glucose release and reduced its uptake by hepatocytes, which caused the postprandial hyperglycemia and insulin resistance [234,235]. Therefore, it is not to be excluded that restoration of the brain dopaminergic systems due to BC treatment prevents the weakening of the hypothalamus, and interrupts the chain of pathological changes in the CNS and in the periphery.

The ability of BC to control the levels of biogenic amines in the suprachiasmatic nucleus of hypothalamus and the circadian rhythm of secretion of 5-HT and other neurotransmitters is also of great importance in the regulation of metabolic processes in diabetic pathology. The treatment of diabetic hamsters with BC provoked the 12 h shift of the maximum daily concentrations of 5-HT and its metabolite, 5-hydroxyindole acetic acid, which, therefore, were observed in the dark period. The increase of hypothalamic content of 5-HT and its metabolites at the nighttime to values typical of healthy animals led to restoration of functions of the brain signaling network [236]. As a result, functional state of diabetic animals improved, insulin sensitivity increased and the elevated levels of glucose and free fatty acids decreased.

It has been accepted for a long time that functions of the cardiovascular system are dependent on the brain signaling systems, the dopaminergic in particular. The decrease in the activity of the dopaminergic system in the CNS led to hyperactivation of the myocardial sympathetic nervous system and contributed significantly to the development of cardiovascular diseases [222]. Other causes of dysfunctions of the cardiovascular system in T2DM are the disturbances of carbohydrate and lipid metabolism, lipotoxicity, oxidative stress, insulin resistance and the elevated levels of proinflammatory factors, all causing damage of vascular endothelial cells, triggering inflammatory processes in them and inducing the atherosclerotic plaque formation [237]. The treatment of diabetic patients with BC is one of the most perspective approaches to prevent vascular pathology [232,238]. As shown by the results of clinical trials and experiments with hypertensive rats, dysfunctions in the cardiovascular system can be a good reason to use BC in the treatment of T2DM and MS [212,232]. In addition, BC prevents the abnormalities in the excretory system of diabetic patients and in the case of chronic kidney diseases slows down their progress [239].

We demonstrated that the 2-month BC treatment of rats with HFD-induced T2DM resulted in the restoration of glycometabolic parameters and improved insulin sensitivity [226,240]. Along with this, it was found that BC partially restored the AC inhibitory effects of 5-nonyloxytryptamine and somatostatin in the brain of diabetic rats [226], which may be expected due to the relationship between DA_2_R and the somatostatin and serotonin signaling systems in the CNS. The BC treatment normalized the adrenergic signaling and regulatory effects of relaxin and somatostatin in the myocardium, and restored the AC sensitivity to gonadotropin in the Leydig cells, indicating a broad therapeutic potential of BC in T2DM therapy [241].

As far as changes in G_s_ protein-coupled DA_1_R are concerned, this problem was studied by the other authors and the data they obtained are available now [242]. Co-administration of DA_1_R and DA_2_R agonists into *ob*/*ob* mice and rats with T1DM suppressed appetite and hyperphagia more effectively than the treatment with DA_2_R agonist alone [243,244]. The anorectic effect of DAR agonists is due to their ability to inhibit the expression of NPY and its receptors, elevated in DM. As a result, agonists of DAR normalized the NPY signaling pathways in hypothalamic neurons and restored the feeding behavior dependent on them. The suppression of appetite by DAR agonists in DM, as compared with healthy animals, required their higher doses and was less effective, which may have been due to the weakening of the brain dopaminergic system and the increased activity of hypothalamic neurons mediating the NPY effects. Nevertheless, there are grounds to expect that in future the DA_1_R agonists in combination with DA_2_R will find application in the treatment of T2DM and MS.

## Serotonin signaling system

The 5-HT, an important neurotransmitter, acting through the brain serotoninergic signaling system regulates feeding behavior, motor activity, pain, sleep, mood, sexual activity, depression, anxiety, aggression and learning. Brain 5-HT is involved in the control of the cardiovascular, reproductive and endocrine systems, and regulates the synthesis and secretion of insulin and other hormones by the pancreas [245,246]. The regulatory effects of 5-HT are implemented through 15 subtypes of metabotropic 5-HTR and three subtypes of ionotropic 5-HT_3_R. The metabotropiс 5-HTR, depending on the type of G-protein interacting with them, are subdivided into four groups: the G_i/o_-coupled 5-HT_1A_R, 5-HT_1B_R, 5-HT_1D_R, 5-HT_1E_R and 5-HT_1F_R; the G_q/11_-coupled 5-HT_2A_R, 5-HT_2B_R and 5-HT_2C_R; the G_s_-coupled 5-HT_4_R, 5-HT_6_R and 5-HT_7_R and the 5-HT_5A_R and 5-HT_5B_R interacted with both G_q/11_- and G_i/o_-proteins [247,248].

In the brain, 5-HT is responsible for the development, differentiation and regeneration of neuronal cells, whereby the abnormalities in 5-HT signaling leads to the impaired synaptic plasticity, imbalance of neuronal network, neurodegenerative changes and eventually to psychiatric diseases [249,250]. The disturbances in the brain 5-HT signaling also result in eating disorders, metabolic dysfunctions and the decrease of insulin sensitivity, and can be a trigger for obesity, MS and T2DM [251].

It was shown that in the CNS of patients with DM2 and MS the free level of tryptophan, a precursor of 5-HT, and the ratio of free/total tryptophan were decreased [252–255]. The decrease of 5-HT concentration and the changes of 5-HT metabolism due to alteration of the activity of tryptophan-5-hydroxylase-2, the rate-limiting enzyme of 5-HT biosynthesis, led to abnormalities in the brain 5-HT signaling, to alterations of the number and affinity of 5-HTR, and to impairment of 5-HT-mediated regulation of peripheral metabolism and insulin sensitivity [254–257].

The data on 5-HT deficiency in the brain of diabetic patients allow the assumption that the increase of serotonin level in the CNS is an appropriate method to improve feeding behavior, energy expenditure, glycemic control and insulin sensitivity impaired in diabetic pathology. This is confirmed by the results of treatment of patients with depression using selective serotonin reuptake inhibitors (SSRIs). Treatment with fluoxetine decreased body weight, normalized the glucose level, reduced the blood level of glycosylated hemoglobin, improved the insulin sensitivity, as well as prevented neurological disorders [34,40,258–262]. We showed that the long-term treatment of female rats with neonatal model of T2DM using intranasally administered 5-HT improved metabolic parameters and cognitive functions, and restored the insulin sensitivity [34]. According to the other authors, the treatment of obese glucose-intolerant mice with selective 5-HT_2C_R-agonist BVT.X significantly improved glucose tolerance and reduced the plasma insulin level; these effects were observed at the BVT.X concentration ineffective in respect of feeding behavior, energy expenditure, body weight and locomotor activity [40]. The pronounced effect of the 5-HT_2C_R agonist on insulin resistance was due to the restoration of hypothalamic 5-HT_2C_R signaling decreased in MS and DM2. There is a lot of evidences that attenuation of 5-HT_2C_R signaling leads to hyperphagia, disturbed energy expenditure, obesity, reduced insulin sensitivity, and the hypothalamic melanocortin system closely linked to 5-HT_2C_R is involved in these metabolic and behavioral changes [40,42,263]. The MC_4_R and 5-HT_2C_R are co-localized in neurons of the ARC of the hypothalamus and both are directly involved in the effects of 5-HT_2C_R-agonists on insulin sensitivity [40]. The 5-HT_1_R also participate in the regulation of feeding behavior and insulin sensitivity. The activation of 5-HT_1B_R by *m*-chlorophenylpiperazine, a mixed agonist of 5-HT_1B_R and 5-HT_2C_R induced a decrease of appetite and partially restored insulin sensitivity in mice lacking 5-HT_2C_R. The injection of the 5-HT_1A_R-agonist 8-hydroxy-2-(di-*n*-propylamino)tetralin (8-OH-DPAT) into the PVN and the anterior medial nucleus accumbens, and of the 5-HT_1B_R-agonist CP-93,129 into the parabrachial nucleus of the pons caused a significant decrease of food intake and changed the dietary preferences [264–267]. This indicates that the interaction between the 5-HT_1_R-, 5-HT_2C_R- and MC_4_R-dependent pathways in the hypothalamic and the other brain areas contributes greatly to the control of the food intake, glucose tolerance and insulin sensitivity [42].

All this gave grounds to say that the elevation of brain serotonin level and the restoration of serotoninergic neurotransmission may provide optimization of the metabolic control in T2DM. On the other hand, there are clinical data showing that chronic administration of SSRI into nondiabetic patients can lead to insulin resistance and lipid metabolism disorders [268,269]. These data may be explained by the fact that a long-term increase of brain serotonin level above norm induces hyperactivation of serotonin signaling, resulting in the abnormalities of feeding behavior, energy expenditure and insulin sensitivity, and, in addition, leads to the changes in the circadian rhythm. The latter directly changes the functions of the hypothalamic-pituitary-adrenal axis and leads to disturbances in synchronization of the release of serotonin and other neurotransmitters, DA in particular [28,29]. Besides, the role of different types of 5-HTR in the control of energy homeostasis differs significantly [267,270,271]. It was shown that the bilateral infusions of the 5-HT_6_R-agonist EMD 386088 into the nucleus accumbens caused the increase of food intake in both food-restricted rats and animals on fat/sucrose diet, while 5-HT_1A_R-agonist 8-OH-DPAT suppressed the feeding behavior in the same brain area [267,272]. These results indicate that 5-HT_1_R and 5-HT_6_R in the nucleus accumbens regulate the appetitive components of food-directed motivation in different ways and play a different role in modulating the food consumption.

Since the changes in the brain 5-HT signaling in T2DM and MS are receptor-and brain area-specific [273,274], this specificity should be taken into account in developing the approaches for their correction. In future, it will be possible to improve the brain 5-HT signaling in T2DM and MS by using selective agonists of the 5-HT_1A/1B_R and 5-HT_2C_R, by optimizing SSRI therapy, and by developing the approaches based on the co-administration of 5-HTR agonists and other regulators of CNS.

## Melanocortin signaling system

The brain melanocortin signaling system plays an important role in regulation of blood glucose, insulin sensitivity, feeding behavior and lipid metabolism [275]. The sensor components of this system are MC_3_R and MC_4_R activated by α-MSH and other peptides of the melanocortin family that are generated as a result of proteolytic cleavage of POMC. The α-MSH (fragment 138–150) binds to all types of MCR, while β-MSH (217–234) and γ-MSH (77–87) specifically bind to MC_4_R and MC_3_R, respectively. Both types of MR are widely present in the hypothalamus, thalamus, brain stem and cortex, indicating their involvement in regulation of many autonomic and neuroendocrine functions. The activation of MC_3_R, autoreceptors located in the presynaptic membrane, leads to reduction of the activity of POMC neurons and to a decrease of the MC_3_R and MC_4_R signaling pathways, thereby MC_3_R agonists function as negative regulators of the melanocortin signaling. On the other hand, the binding of G_s_ protein-coupled MC_3_R and MC_4_R with their agonists leads to activation of melanocortin-sensitive AC signaling system, and so the regulatory effects of MC_4_R and MC_3_R can be potentiated [276,277]. The MC_4_R and MC_3_R agonists through the 3-phosphoinositide pathway increase the intracellular calcium concentration and activate the protein kinases PI3K and ERK1/2 [278,279]. Along with melanocortin peptides, AGRP/NPY-neurons of the ARC of the hypothalamus produce AGRP with MC_3_R/MC_4_R antagonistic activity [280]. The AGRP inhibits regulatory effects of melanocortins, and through MC_4_R activates G protein-independent arrestin signaling cascades [281].

As the hypothalamic melanocortin signaling has an important role in regulation of glucose metabolism and insulin sensitivity, the decrease of its activity leads to hyperphagia, metabolic disorders, insulin resistance, and eventually to severe obesity, MS and T2DM [282–284]. Mice lacking MC_4_R had the elevated insulin level and the decreased insulin sensitivity even before manifestation of hyperphagia and obesity [282,285]. These mice had obesity and metabolic dysfunctions similar to those in mice with agouti (yellow obese) syndrome characterized by increased AGRP expression [286,287]. The administration of high doses of AGRP and synthetic MC_4_R antagonists into healthy mice enhanced appetite and led to insulin resistance [33]. The MC_4_R agonists (α-MSH, melanotan-II), on the contrary, reduced food intake, normalized the glucose and insulin levels and energy metabolism [33,282–284]. In patients with MS and T2DM the changes in MC_4_R signaling cascades and the mutations in gene encoding MC_4_R were identified [288–292]. The patients with the mutations had severe obesity, hyperphagia and hyperinsulinemia, and in homozygotes these symptoms were more pronounced than in heterozygotes [288].

The melanocortin signaling system modulated the regulatory effect of insulin on glucose homeostasis through two mechanisms, suppression of glucose production and enhanced glucose disposal in the liver. This is the basis of melanocortins-mediated control of insulin resistance. It has been established that central effects of melanocortin peptides on insulin sensitivity are tissue specific. The blocking of MC_4_R in the hypothalamus, on the one hand, increased insulin sensitivity in the white adipose tissue and enhanced glucose uptake in adipocytes, and on the other hand, reduced insulin sensitivity in the skeletal muscles, causing an increase of insulin level in this tissue [284]. The influence of α-MSH on insulin sensitivity also depended on the target tissue. The activation of MC_4_R in the CNS led to the increase of insulin sensitivity in many tissues, while the activation of the melanocortin system in the periphery, on the contrary, usually induced the decrease in insulin sensitivity [293], which is very important in the light of etiology and pathogenesis of metabolic disorders.

The hypothalamic melanocortin system regulates the lipid metabolism and body weight more quickly and more efficiently than the other brain signaling systems [284]. An important role in this belongs to leptin that controls the production of POMC and AGRP [294]. The increase of brain leptin level led to the increase of expression of POMC and MC_4_R genes, and reduced expression of AGRP gene [295]. As has been said above, the brain melanocortin system mediated many of the antidiabetic effects of leptin [296]. Meanwhile, the effect due to activation of this system did not coincide with those of leptin, which prompts participation of the other brain signaling systems in the central effects of this hormone [297].

The decrease of activity of hypothalamic melanocortin system can induce dysfunctions in the brain integrative network and provoke the neurodegenerative changes, which leads to AD and other neurological disorders [298–300]. The restoration of MC_4_R signaling had, in turn, neuroprotective and neurotrophic effects, improved adaptive neuronal plasticity, stimulated the regeneration of neuronal and glial cells and inhibited production and activity of proapoptotic and proinflammatory factors [298,300,301–303]. The treatment of gerbil with ischemic stroke with the MC_4_R agonist Nle^4^,D-Phe^7^-α-MSH decreased the activity of inflammatory and apoptotic cascades in the hippocampus, preventing the severe DNA damage and delayed neuronal death, and reduced hippocampus injuries even when delayed up to 9 h after ischemia [298]. Due to the MC_4_R-mediated protection of hippocampal neurons, the 12-day treatment of animals with Nle^4^,D-Phe^7^-α-MSH prevented the decrease of the spatial memory and the learning caused by ischemic stroke. These data indicate the participation of MC_4_R in the neurotrophic action of melanocortin peptides, resulting in the stimulation of axon growth and regeneration of damaged neurons [298]. The MC_4_R agonists also exerted potent anti-inflammatory effects by antagonizing the effect of interleukin 1β and other proinflammatory cytokines, inducing the impairment of memory consolidation, whose activity in the CNS increases substantially the diabetic pathology [302,303].

It was shown the administration of melanocortins induced the neuroprotection in transgenic mice Tg2576 having the cognitive deficit and neurodegenerative changes associated with a low level of the synaptic activity-dependent gene *Zif268*, the experimental model of AD [304]. The treatment of transgenic animals with Nle^4^,D-Phe^7^-α-MSH decreased neuronal loss, reduced the level of β-amyloid peptide deposit in the cerebral cortex and hippocampus, improved cognitive functions and restored the expression of the gene *Zif268* in the hippocampus. Therefore, the MC_4_R stimulation by melanocortins is capable of counteracting the cognitive decline in AD and other neurodegenerative diseases through the induction of neuroprotection and the improvement of synaptic transmission [300,304]. There are grounds to suggest that the changes in the brain melanocortin system are one of the main causes of neurodegenerative processes contributing to the impairment of hippocampal plasticity, cognitive deficit and metabolic dysfunctions in T2DM and MS.

To define the role of the brain melanocortin system in etiology and pathogenesis of MS and T2DM, of great importance is the study of experimental models of autoimmune diseases induced by immunization of animals with peptides corresponding to the extracellular loops of MCR. In 2008, Hofbauer and colleagues showed that immunization of rats with peptide corresponding to the N-terminal extracellular portion of MC_4_R led to the increase of body weight and the levels of triglyceride and insulin [305]. Antibodies to peptides acted as the partial agonists and by their pharmacological properties were similar to AGRP and MC_4_R antagonists. The treatment of rats with peptide corresponding to the third extracellular loop of MC_3_R caused the increase in body weight and blood pressure, the elevated levels of insulin, glucose and leptin, all typical of MS and T2DM. In the *in vitro* experiments antibody directed against MC_3_R-peptide acted as noncompetitive antagonists and reduced the AC stimulatory effect of α-MSH [306]. We studied long-term MC_4_R deficiency on rats using the autoimmune model; the animals were immunized with BSA-conjugate of peptide K-[TSLHLWNRSSHGLHG^11–25^]-A corresponding to the region 11–25 of the N-terminal portion of MC_4_R. Nine–thirteen months after the first immunization the animals had weight gain, impaired glucose tolerance, insulin resistance and dyslipidemia. The MC_4_R activity in the hypothalamus was decreased, which was illustrated by weakening of the stimulating effects of MC_4_R-agonists on AC activity [307,308]. To create the model of MC_3_R deficiency, rats were immunized with BSA-conjugate of peptide A-[PTNPYCICTTAH^269–280^]-A corresponding to the third extracellular loop of MC_3_R, and 13 months later the immunized animals had dyslipidemia, decreased body weight, but increased mass of abdominal fat, which indicated changes in the fat/muscle weight ratio and abnormalities in the lipid metabolism [240]. Thus, long-term antibody-induced shutdown of MC_4_R and MC_3_R may be interpreted as indicating the possible role of the melanocortin signaling system in the development of MS and T2DM.

Nowadays, the use of MC_4_R-agonists in the treatment of metabolic disorders is confined to experimental models of obesity and MS [33,283,284,309–312]. This is due to the lack of available and selective MC_4_R-agonists, and the pleiotropy of their action, which leads to a large number of side effects [313]. The preference is generally given to nonselective agonist melanotan-II, which reduces body weight and appetite and improves the carbohydrate and lipid metabolism [309–311]. But the use of melanotan-II for a long time led to severe side effects, so this drug is not to be recommended for correction of metabolic disorders. Currently, new MC_4_R-agonists continue to come on scene, but they have not been used in clinic yet.

The most effective among them are α-MSH analogs modified by fatty acid radical at the N-terminus and containing the d-Phe-Arg-Trp motif responsible for pharmacological activity of α-MSH and the N-terminal segment Ser-Tyr-Ser responsible for selectivity of interaction with the receptor [314]. The lipidated α-MSH analog MC4-NN1–0182 had a prolonged effect and interacted with MC_4_R with a high selectivity and affinity. The 3-week treatment of DIO-rats Sprague–Dawley and 8-week treatment of minipigs with MC4-NN1–0182 led to the weight loss, and a single treatment of rats induced the increase of insulin sensitivity and glucose utilization [312]. Highly-selective MC_4_R agonists BIM-22493 (RM-493) that cross the BBB when administered peripherally improved obesity, hyperinsulinemia and fatty liver diseases in DIO-mice [315,316]. The 8-week treatment of obese rhesus macaques with this drug resulted in the decrease of food intake, body weight and fat mass, and in the improved glucose tolerance [316]. Unexpected was the fact that the increased sensitivity to insulin in BIM-22493-treated animals was maintained for at least 4 weeks after the end of treatment. Note that even a prolonged treatment of mice and monkeys with BIM-22493 had no effect on the cardiovascular system and blood pressure, the adverse effects typical of nonselective MCR agonists [315,316]. Another selective MC_4_R agonist AZD2820, a cyclic analog of α-MSH, prevented the increase in body weight in mice which received HFD after fasting [317].

The effectiveness of MC_4_R agonists can be greatly enhanced when they are combined with the agonists of GLP-1 receptor, which is now widely used in the treatment of MS and T2DM [318]. The co-administration of BIM-22493 and liraglutide, a stable GLP-1 receptor agonist, into DIO-mice increased insulin sensitivity, decreased body and fat mass and improved energy expenditure much more effectively than monotherapy [319]. Unlike monotherapy, in the case of co-administration of BIM-22493 and liraglutide the expression of *Mc4r* and *Glp-1r* genes in hypothalamic neurons increased, indicating the absence of MC_4_R and GLP-1 receptor desensitization. As there are many activators of GLP-1 receptor, which include not only stable GLP-1 receptor agonists, but also inhibitors of the enzyme dipeptidyl peptidase-4 destroying endogenous GLP-1, there are many opportunities to further optimize the therapy directed to simultaneous activation of MC_4_R and GLP-1 receptor.

## Glucagon-like peptide-1 & its signaling

The GLP-1 belonging to the incretin family activates the proliferation of pancreatic β-cells and increases the secretion of insulin, regulating the glucose and insulin levels and insulin resistance. The GLP-1 is secreted primarily by enteroendocrine L cells of the small intestine, but a small amount of hormone is produced in the CNS [320]. In patients with MS and T2DM, GLP-1 restored insulin sensitivity, improved glycemic control, reduced the oxidative stress and prevented the disturbances in the cardiovascular system, which makes GLP-1 and its analogs the promising drugs to treat these diseases [321–324]. The effects of GLP-1 were realized by the peripheral mechanisms based on its influence on pancreatic β-cells, and through the central pathways where GLP-1 acts as a neurotransmitter and growth factor [325]. This was confirmed by the evidence that GLP-1 had neuroprotective and neurotrophic effects on neuronal cells and influenced the synaptic plasticity [320,326–328].

The effects of GLP-1 on neuronal activity are due to its binding with GLP-1 receptors widely distributed in the brain. The binding of GLP-1 receptor with hormone provokes the activation of AC and cascade of MAPKs, and also modulates the activity of Ca^2+^ channels [329]. In MS and T2DM, the level of GLP-1, its secretion in response to food intake and the activity of GLP-1 signaling system are reduced, leading to the disturbances of insulin sensitivity, energy metabolism and neurogenesis [324,330,331].

The data are available suggesting that functional impairments in neuronal cells due to weakening of the GLP-1 system are likely to alter the activity of other neurotransmitter systems, primarily dopaminergic, which causes disintegration of the brain signaling network and enhances insulin resistance, dyslipidemia and oxidative stress, and exacerbates the metabolic disorders. Consequently, restoration of the brain GLP-1 signaling system can be used to correct the metabolic and functional abnormalities MS and T2DM [332]. The high potential of this approach is based on a large number of pharmacological agents, regulators of this system, including the proteolysis-resistant GLP-1 analogs (exendin-4, liraglutide and others) and the inhibitors of DPP-4 hydrolyzing GLP-1 [333,334]. Now the DDP-4 inhibitors are widely used to treat T2DM, due to their ability to restore the lipid metabolism and to improve the insulin sensitivity and glycemic control [335–338]. Additionally, these inhibitors exert the neurotrophic and neuroprotective effects on neuronal cells and prevent the DM-associated cognitive dysfunction and neurological disorders [339–341].

Most frequently in the treatment of patients with T2DM were used exendin-4 and liraglutide; they improved glycemic control, reduced insulin resistance, decreased excessive body weight and prevented neurodegenerative changes [327,333,334,342,343]. These drugs easily penetrate the BBB, which made them effective both in peripheral and central administration. The 4–10-week treatment of *ob*/*ob* and *db*/*db* mice and DIO-mice with exendin-4 and liraglutide increased by 100–150% the rate of neuronal cells proliferation and stimulated their differentiation, while exendin (9–36), antagonist of GLP-1 receptor, inhibited these processes [325]. Exendin-4, liraglutide and other GLP-1 receptor agonists reduced the amyloid plaque formation in patients with T2DM, MS and AD, the same refers to mice with insulin resistance and T1DM. Preventing the neurodegenerative processes, they also improved central control of peripheral metabolism [344–346]. Another target of GLP-1 in the brain is the dopaminergic system. The GLP-1 and its analogs upregulated the expression of tyrosine hydroxylase catalyzing the conversion of tyrosine into L-DOPA, DA precursor, in addition, they suppressed the inhibitory effect of proinflammatory and neurotoxic agents on dopaminergic neurons [347–349]. It was found that exendin-4 suppressed the inflammatory processes in neurons of diabetic mice with experimental cerebral ischemia by inhibiting the expression of proinflammatory cytokines [327]. These data indicate that GLP-1 and its analogs are good candidates to improve insulin sensitivity and glycemic control and to prevent the neurodegenerative processes in the brain in MS and T2DM.

## Conclusions & future perspectives

In prediabetes, MS and T2DM the functional state and the interaction between the brain signaling systems undergo significant changes. The degree of these changes depends on the severity of insulin resistance, dysfunctions of pancreatic β-cells, oxidative stress and lipotoxicity, and augments during transition from prediabetes to overt T2DM. At each stage of development of diabetic pathology the alterations in hormonal and neurotransmitter systems grow in number, which eventually leads to malfunctions of the integrative signaling system of the brain. The identification of molecular disturbances in the brain signaling systems regulated by insulin, IGF-1, leptin, DA, 5-HT, melanocortins, GLP-1 and other hormones and neurotransmitters is a reliable way to make the early diagnostics concerning the “weakness and flaws in the CNS” contributing to the development of MS and T2DM, and to develop the new approaches to their treatment and prevention based on restoration of these systems (Table 1). Due to a close link between the brain signaling systems, normalization of the functions of one system can lead to restoration of the others. This points to prospects for comprehensive approach to be used in the treatment of MS and T2DM based on coordinated regulation of some signaling systems. The strategy of correction of the brain signaling systems must be developed taking into account the hormonal and functional disturbances in the certain brain areas, and also etiology, pathogenesis and severity of metabolic disorders. In prediabetes and the early T2DM, hormonal alterations in the brain are still reversible and can involve one or more signaling systems, while furthermore, they were identified in many of the systems, which requires the use of various treatment strategies. It is important to use combination of approaches restoring signal transduction in the CNS and those focusing on reduction of hyperglycemia, oxidative stress and lipotoxicity, the most important factors causing abnormalities in the brain signaling systems in MS and T2DM.

**Table 1.** **The approach to the improvement of brain signaling systems regulated by hormones and neuromediators in Type 2 diabetes mellitus and metabolic syndrome.**

**Approach/strategy**	**Current state**	**Future prospects**
The increase of brain levels of hormones and neuromediators due to their intranasal administration, the use of the reuptake inhibitors and the BBB-penetrating analogs of hormonal agents	The use of intranasal insulin to correct the neurodegenerative disorders and in experimental T2DM [117,119,120,122,124]; the use of BBB-penetrating analogs of leptin in experimental obesity and T2DM [185,206,207]; the use of SSRI and intranasal 5-HT to treat diabetic patients and in experimental conditions [34,258–262]	The development of effective approaches to intranasal and inhalation routes of administration of leptin, melanocortins and other regulators of the brain signaling; the development of nanoparticles for nasal delivery of hormonal agents; the development of the BBB-penetrating conjugates of hormonal agents with chitosan, PEG and other macromolecular carriers
The use of highly selective agonists/antagonists of hormonal receptors, which selectively regulate specific signaling pathways and influence a certain type of neuronal cells	The use of DA_2_R-agonist bromocriptine to improve glucose tolerance and cardiovascular functions in diabetic patients [210,212,215–223]; the use of leptin-derived peptide ([D-Leu-4]-OB-3) in experimental models of T2DM [198–200]; the use of the 5-HT_2C_R-agonists to improve glucose tolerance in experimental model of T2DM [40]; the use of the MC_4_R agonists (Nle^4^,D-Phe^7^-α-MSH, BIM-22493, AZD2820, etc.) to improve feeding behavior, insulin sensitivity and cognitive functions in experimental models of metabolic disorders [298,300,304,310,312–317]	The development of new classes of highly selective agonists of the leptin, melanocortin and GLP-1 receptors, including the low-molecular-weight compounds, and the development of biased (functionally selective) agonists of 5-HTR and DAR
The application of hormones/neuromediators analogs resistant to the degradation	The use of proteolysis-resistant GLP-1 analogs (exendin-4, liraglutide, etc.) to improve feeding behavior, glycemic control and insulin sensitivity, and to prevent neurodegenerative changes in patients with T2DM and in experimental models of metabolic disorders [333–335,342,343,345,346]	The use of proteolysis-resistant GLP-1 analogs to treat patients with T2DM and MS; the development of the proteolysis-resistant leptin and melanocortin analogs by the chemical modification, the amino acid substitutions and the synthesis of truncated analogs
The use of agents that enhance synthesis and secretion of hormones and neuromediators and prevent their degradation in the CNS	The use of dipeptidyl peptidase-4 inhibitors to restore the insulin sensitivity and the metabolic processes in patients with MS and T2DM [333–338]; the use of IDE inhibitors to improve glucose tolerance in experimental metabolic disorders [128,129]	The development of therapeutic approaches to effective clinical use of dipeptidyl peptidase-4 inhibitors alone and in the combination with other CNS regulators and antidiabetic drugs; the search of IDE inhibitors suitable for clinic application; the search of pharmacological regulators of the enzymes responsible for brain synthesis of DA, 5-HT and other neuromediators
The application of regulators that act at the postreceptor stages of neuronal signal transduction	The use of PTP1B inhibitors (Trodusquemine, Claramine) to improve insulin and leptin sensitivity in experimental metabolic disorders [142–144,204]	The search of new highly selective regulators of phosphatases, protein kinases, cAMP-phosphodiesterases, and other proteins controlling the postreceptor stages of insulin, IGF-1 and leptin signaling, and their use in clinical endocrinology
The coordinated regulation of two or more brain signaling systems	Administration of leptin with insulin, amylin, cholecystokinin and GLP-1 analog to enhance their effects on the animals with experimental metabolic disorders and in clinical trials [186–193]; administration of bromocryptine with insulin, metformin, glipizide and pioglitazone to synergize their effects in patients with T2DM and in experimental diabetes [219,222,225,228]; co-administration of MC_4_R agonists and GLP-1 analogs to improve insulin sensitivity and energy expenditure in experimental animals much more effectively than monotherapy [319]	The search of the most effective approaches to co-administration of hormonal regulators of the brain signaling systems in diabetic pathology; the optimization of the schemes for clinical application of synergistically acting hormones, including the decrease of their effective doses and the duration of treatment

**Executive summary**

**Background**

The changes and abnormalities in the brain signaling systems regulated by hormones and neuromediators have an important role in etiology and pathogenesis of Type 2 diabetes mellitus (T2DM), metabolic syndrome (MS) and their complications. They can be the cause of these diseases, so the restoration of their activity at the early stages of T2DM and MS is one of the most promising ways to treat and prevent their severe forms.

**Brain insulin/IGF-1 signaling**

The data on structural-functional organization of the brain signaling systems regulated by insulin and IGF-1, and on the important role of the impairment in these systems in etiology and pathogenesis of obesity, prediabetes and T2DM are presented. The approaches to the restoration of brain insulin/IGF-1 signaling in metabolic disorders, including the elevation of insulin level in the CNS due to its intranasal administration and the use of selective inhibitors of phosphatase PTP1B to prevent central insulin resistance, are discussed.

**Leptin signaling system**

The evidence that leptin deficiency and leptin resistance in the hypothalamus result in imbalance of neuronal interactions, deregulation of peripheral metabolism and insulin resistance, which leads to metabolic, neuroendocrine and neurological disorders is presented. Many approaches to restore the brain leptin signaling, including leptin analogs penetrating the blood–brain barrier, activators of the postreceptor stages of leptin signaling, and co-administration of leptin and other hormones are considered.

**Dopamine signaling system**

The dysfunctions of dopamine signaling system, leading to insulin resistance and abnormalities of carbohydrate and lipid metabolism, and the great therapeutic potential of D_2_-agonist bromocryptine that improves the glycemic control and prevents cardiovascular disorders in T2DM and MS are discussed.

**Serotonin signaling system**

The involvement of the impaired brain serotonin signaling in eating disorders, metabolic dysfunctions and insulin resistance, and possible ways of their restoration are considered.

**Melanocortin signaling system**

The key role of changes in hypothalamic melanocortin signaling system in the development of hyperphagia, energy imbalance and insulin resistance, and the pharmacological approaches to improve this system, such as the use of selective agonists of type 4 melanocortin receptor and the co-administration of melanocortins and GLP-1 analogs are discussed.

**Glucagon-like peptide-1 & its signaling**

The interrelation between the functional activity of GLP-1 signaling and the brain and peripheral insulin sensitivity in T2DM and MS, and the therapeutic potential of the proteolysis-resistant GLP-1 analogs and the inhibitors of dipeptidyl peptidase-4 are considered.

**Conclusions & perspectives**

The approaches now in use to improve the brain signaling in diabetic pathology are summarized, and the prospects of their development are suggested.
